# Immediate and non-immediate allergic reactions to amoxicillin present a diagnostic dilemma: a case series

**DOI:** 10.1186/s13256-016-0801-2

**Published:** 2016-01-18

**Authors:** Caroline Weisser, Moshe Ben-Shoshan

**Affiliations:** 1 Division of Pediatrics, Department of Pediatrics, Janeway Children’s Hospital, St. John’s, Newfoundland Canada; 2 Division of Allergy and Clinical Immunology, Department of Pediatrics, Montreal Children’s Hospital, Montreal, Quebec Canada

**Keywords:** Allergic reactions, Amoxicillin, Oral drug challenge

## Abstract

**Background:**

Allergic reactions to amoxicillin are very common occurrences in the pediatric age group; however, onset of symptoms can present a diagnostic dilemma.

**Case presentation:**

We present a case series that describes three children (8-year-old white girl, 2-year-old white boy and 14-month-old Chinese boy) who presented with varied onset of allergic reactions to amoxicillin, specifically immediate (within the first hour after exposure) and non-immediate onset. One child developed immediate onset allergy to oral challenge with amoxicillin although his clinical history was evident for non-immediate onset allergy to amoxicillin. He was the only case that had a positive skin test to penicillin. Two other children presented with reactions toward the end of their treatment course of amoxicillin, yet one patient developed immediate onset allergy while the other patient developed non-immediate onset allergy after challenge.

**Conclusions:**

This case series demonstrates diagnostic challenges facing physicians assessing allergic reactions to amoxicillin. As onset of reactions can dictate severity and pathogenic type of allergy, a thorough clinical history and subsequent appropriate diagnostic testing including medication challenge can help establish the diagnosis.

## Background

Amoxicillin is a commonly prescribed antibiotic for treatment of community-acquired bacterial infections in children [1]. Given that it is a first-line treatment for otitis media and sinusitis, and given the high frequency of viral-induced exanthemas including hives in this age group [2, 3], it is not surprising that rashes developing during the course of amoxicillin treatment are frequently reported [1, 4]. Furthermore, up to 70 % of patients receiving amoxicillin during viral infections, particularly Epstein–Barr virus, are reported to develop a self-limiting maculopapular rash [5]. The estimated incidence of allergy to amoxicillin ranges from 1 to 10 % [2, 4, 6]. However, many cases are diagnosed as allergic reactions without performing appropriate diagnostic tests [1]. A detailed clinical history needs to account for viral exanthemas in the differential diagnosis although the distinction according to history is often challenging.

True allergic reactions to amoxicillin are mediated by the immune system and are classified into immediate (developing within 30 to 60 minutes of drug ingestion) or non-immediate (beyond 1 hour of ingestion) type reactions [6, 7]. Immediate reactions may range in severity from eruptions limited to the skin (hives/angioedema) to reactions involving more than one organ system or hypotension (that is, anaphylaxis) [7]. The risk of fatal anaphylaxis with amoxicillin is not well documented, although the risk with penicillin is estimated at 1 in 100,000 [1]. Non-immediate reactions occur more than 1 hour after ingestion of antibiotic and usually last several days [1]. For the most part, they are mild, self-resolving maculopapular exanthemas or hives [1, 7]. Rarely, non-immediate reactions may present with exfoliative dermatitis, acute generalized exanthematous pustulosis (AGEP), Stevens–Johnson syndrome (SJS), toxic epidermal necrolysis (TEN) and drug reaction with eosinophilia and systemic symptoms (DRESS) [6, 8].

Diagnostic confirmation is done by intradermal testing, in vitro testing or oral challenges to the antibiotic in question and the respective antibiotic family [4]. Drug challenges are considered to be the gold standard in establishing a definitive diagnosis of an allergic reaction to drugs [4, 6, 9]. In these cases, the challenge is begun with one 100th to one tenth of the therapeutic dose and if tolerated over 20 minutes, followed by a full dose with an observation period of 1 hour [6]. Among diagnostic procedures used to confirm the presence of amoxicillin allergy, the oral challenge is considered to have the highest sensitivity although false negative cases and cases of re-sensitization have been described; and 1 week challenges have been suggested to increase sensitivity [10, 11].

It is crucial to differentiate between immediate and non-immediate reactions given their different pathogenic mechanisms and management [6]. The immediate reactions are considered to be immunoglobulin E (IgE)-mediated responses and non-immediate reactions are thought to be T cell mediated [6, 7]. Unfortunately, the pathogenesis of allergic reactions to antibiotics in general and amoxicillin in particular is not well characterized; in addition to IgE and T cell-mediated mechanisms it has been suggested that certain antibiotics can bind non-covalently to antigen-interacting structures, such as the T cell receptor or major histocompatibility complex, and cause a direct stimulation of the immune response. The term p-i concept (or pharmacological interaction with immune receptors) has been coined for the latter [12]. Antibiotics are small-sized molecules that are assumed to be non-immunogenic, and hence numerous hypotheses have been advanced to account for their ability to activate the immune system [13]. It has been suggested that antibiotics have an ability to form conjugates to larger carrier proteins in serum or intracellular space (the hapten hypothesis) that are processed and eventually presented to T lymphocytes [6]. Others suggested that certain antibiotics can bind non-covalently to antigen-interacting structures such as the T cell receptor or major histocompatibility complex, and cause a direct stimulation of the immune response [13]. Given that immediate reactions are considered IgE mediated and may progress to a life-threatening reaction while most non-immediate reactions are considered non-IgE mediated and hence not life threatening, it is important to make the distinction between these two reactions. All immediate reactions should be treated by complete avoidance and in the case of need, drug desensitization [7, 14, 15]. However, studies suggest in the latter, future use of the antibiotic is not an absolute contraindication [16]. It is possible that in case 3 the immediate reaction to challenge was not IgE mediated, but rather an accelerated T cell-mediated reaction; however, the pathogenic mechanisms could not be elucidated at this point and hence strict avoidance was advised.

As the onset of non-immediate allergic reactions is varied and the pathogenesis itself is poorly understood, antibiotic reactions are difficult to diagnose even when a detailed clinical history is evident. We present a case series that describes three children who presented with immediate and non-immediate reactions to amoxicillin (Table 1). These cases demonstrate the challenges associated with the diagnosis and management of amoxicillin-related exanthemas.

**Table 1:** Clinical characteristics and diagnostic test results of cases 1 to 3

Case	Clinical history	Oral challenge	Skin test: Pre-Pen® (benzylpenicilloyl polylysine)
1	8-year-old white girl Treated for uncomplicated pneumonia^a^ Immediate reaction: seventh day of treatment, 15 minutes after ingestion	Immediate reaction: 20 minutes after ingestion of full dose	N/A
2	2-year-old white boy Treated for uncomplicated otitis media^a^ Non-immediate reaction: eighth day of treatment, rash noticed in the morning after he woke up	Non-immediate reaction: 18 hours post-ingestion of full dose	N/A
3	14-month-old Chinese boy Treated for otitis media^a^ Non-immediate reaction: third day of treatment, rash noticed after he awoke from sleep, prior to morning dose	Immediate reaction: 15 minutes post-ingestion of initial dose	Positive 8×16 mm (wheal/erythema)

^a^All cases were being treated with amoxicillin, weight-based dose. *N/A* not applicable

## Case presentation

### Case 1 – Immediate reaction

**Fig. 1:**
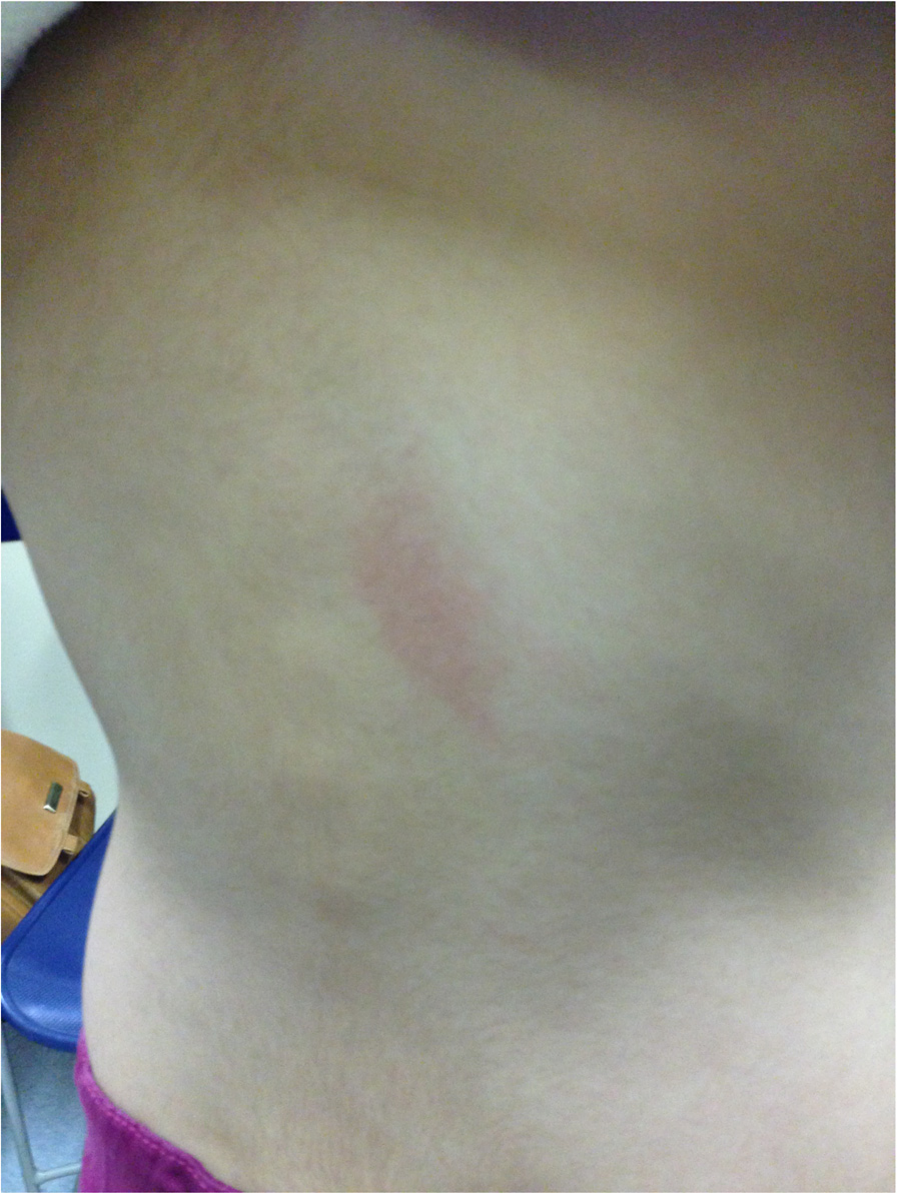
Hives developed on mid-back and right forearm 20 minutes following ingestion of full dose of amoxicillin

A healthy 8-year-old white girl was receiving a standard dose of oral amoxicillin for an uncomplicated pneumonia. On the seventh day of treatment, 15 minutes following her morning dose of amoxicillin, she developed pruritic erythematous plaques that progressed all over her body over the course of the day. There were no systemic signs of anaphylaxis. Amoxicillin was discontinued the same day. The rash resolved after 7 days. It is not known whether this was her first exposure to amoxicillin. She has avoided amoxicillin since then. An intradermal test with Pre-Pen® (benzylpenicilloyl polylysine) was negative. Three months later, she underwent an oral challenge for amoxicillin at our allergy clinic. The oral challenge was positive as she developed hives 20 minutes following ingestion of the full dose (Fig. 1). No other symptoms occurred and the hives resolved after a few hours with no treatment. She was diagnosed with immediate allergy to amoxicillin and advised to avoid amoxicillin and all penicillin family antibiotics.

### Case 2 – Non-immediate reaction

**Fig. 2:**
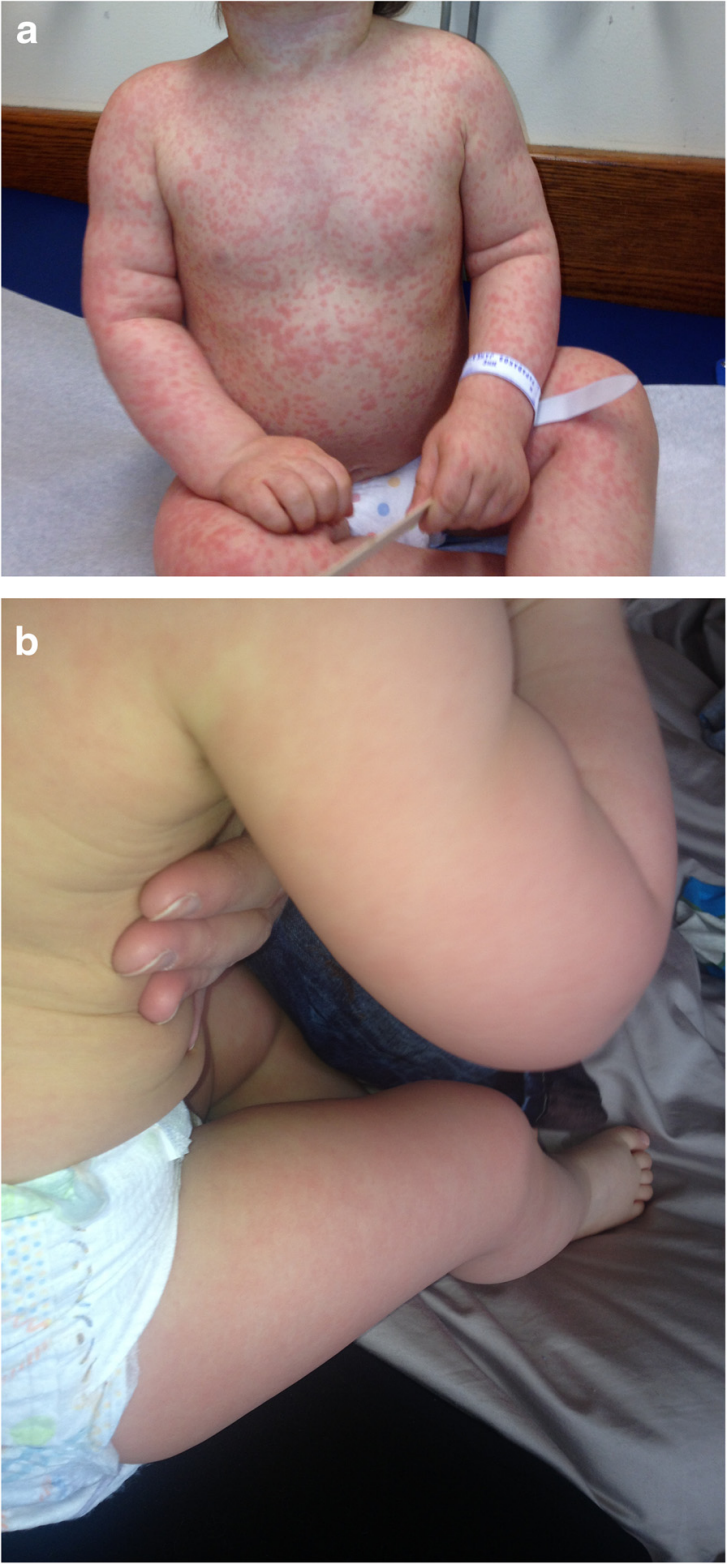
**a.** Erythematous maculopapular rash that coalesced to form large raised plaques developed on day 8 of treatment with amoxicillin. **b.** Erythematous plaques developed on his face, back, thighs and arms 18 hours following oral challenge to amoxicillin

A healthy 2-year-old white boy was receiving a standard dose of oral amoxicillin for an uncomplicated otitis media. On the eighth day of treatment, he developed a maculopapular rash (Fig. 2a) that coalesced to form large raised plaques. The rash was noticed in the morning by parents when he woke up. He was assessed in the emergency room at the local children’s hospital and treated symptomatically with Benadryl (diphenhydramine). His rash lasted 3 to 4 days. He has not taken amoxicillin since then. One month later, he presented at an allergy clinic for an oral challenge to amoxicillin. He was given one tenth of his weight-based dose, observed for 20 minutes and then received the full dose. He had no reactions initially, but approximately 18 hours later he developed non-pruritic erythematous plaques on his face, thighs and arms (Fig. 2b). There were no systemic signs of anaphylaxis. He was given a diagnosis of non-immediate allergy to amoxicillin and advised to avoid amoxicillin and all penicillin family antibiotics.

### Case 3 – Immediate and non-immediate reactions

**Fig. 3:**
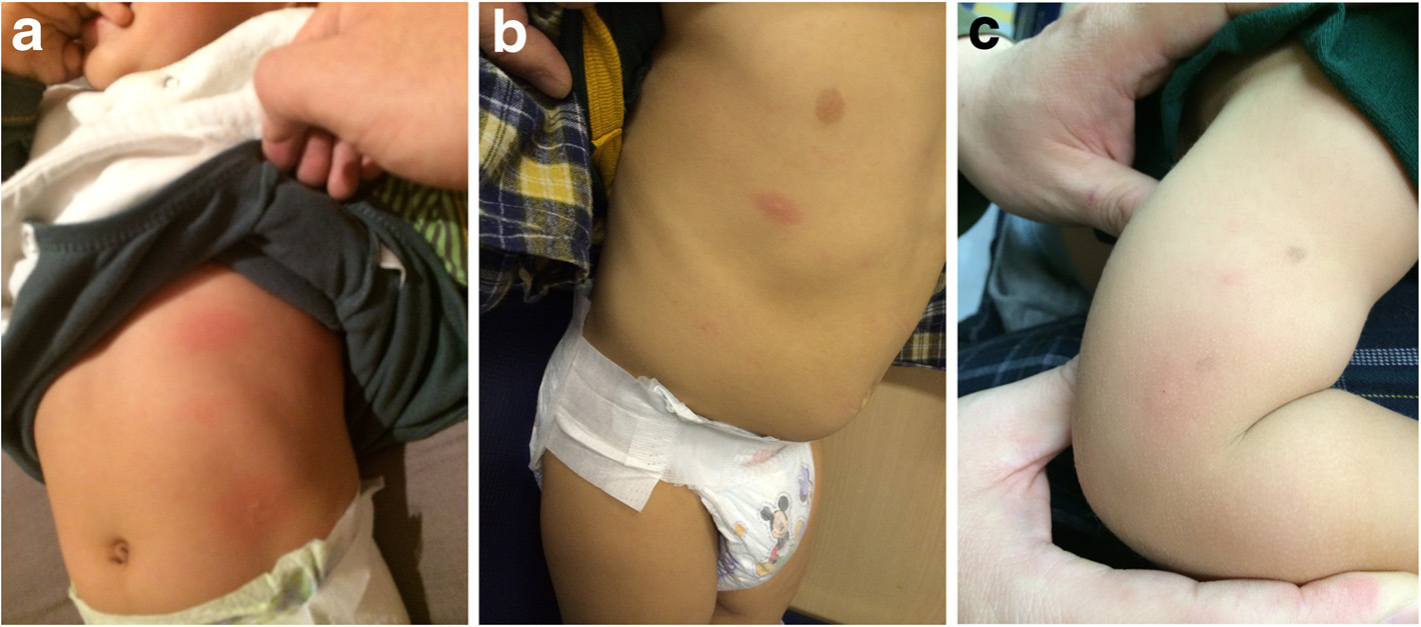
**a.** Hives developed throughout his body on day 3 of treatment with amoxicillin. **b.** Hives developed 15 minutes following ingestion of one tenth of weight-based dose of amoxicillin. **c.** Results of intradermal testing showing wheal size of 8×16 mm (wheal/erythema)

A healthy 14-month-old Chinese boy presented with hives on the third day of amoxicillin treatment for otitis media shortly after he woke up from his sleep and prior to his morning dose (Fig. 3a). There were no systemic signs of anaphylaxis. His rash resolved after 2 to 3 days. One month later, he presented at an allergy clinic for an oral challenge to amoxicillin. The oral challenge was positive because he developed hives 15 minutes (Fig. 3b) following ingestion of one tenth of his weight-based dose of amoxicillin. An intradermal test with Pre-Pen® (benzylpenicilloyl polylysine) was positive (Fig. 3c). He was given a diagnosis of immediate allergy to amoxicillin and advised to avoid amoxicillin and all penicillin family antibiotics.

## Discussion

This case series demonstrates the dilemma in diagnosing immediate versus non-immediate onset allergy to amoxicillin. Our cases demonstrate that children with immediate or non-immediate allergic reactions to amoxicillin may have similar clinical histories. Thus drug challenges may provide a relatively safe and efficient strategy to establish diagnosis in these cases.

Allergic reactions are generally categorized as immediate or non-immediate onset type, with the latter being more frequent [2]. Ponvert *et al*. and Zambonino *et al*. reported that 88 % and 92 % of healthy children were diagnosed with non-immediate allergy following reaction to amoxicillin while 12 % and 8 % were given a diagnosis of immediate allergy to amoxicillin [17, 18]. The authors further demonstrated that children with a likelihood of beta-lactam allergy were more likely to experience early onset and greater severity of disease [17]. Case 3 exemplifies the disparity between a clinical history suggesting a non-immediate onset allergy to amoxicillin and the oral challenge and subsequent intradermal testing establishing the presence of an immediate allergy. Cases 1 and 2 both reacted very late in the course of their amoxicillin treatment (day 7 and 8 respectively), yet case 1 developed immediate onset allergy and case 2 developed non-immediate onset allergy. This demonstrates the variability witnessed in allergic reactions to amoxicillin and potential for diagnostic dilemma without a thorough clinical history and subsequent challenge.

Of interest, no study has researched the likelihood of presenting with non-immediate onset allergy and subsequent positive diagnosis of immediate onset allergy during drug provocation testing. Given that non-immediate reactions are thought to be T cell-mediated responses [6, 7], there are two plausible explanations for the immediate allergy during drug provocation testing in case 3. It is possible that the patient’s immune system developed an IgE-mediated response following the previous non-immediate response. The more plausible explanation could simply be that the parents have failed to notice initial immediate symptoms related to amoxicillin ingestion and hence reported a late occurring reaction.

Finally, our case series demonstrates the limited utility of skin testing in the diagnosis of immediate and non-immediate reactions. This is in keeping with other studies suggesting that although many antibiotics are suspected culprits of immediate reactions, skin tests are either not validated, have a high false-negative rate or are simply not available. For non-immediate reactions, skin tests are even less useful given their high false-negative/positive results [6, 13].

## Conclusions

Although many infections are viral in nature, amoxicillin is a commonly prescribed antibiotic that may trigger immediate and non-immediate allergic reactions in the pediatric age group [1, 4]. Diagnosis of drug allergy can be challenging and an oral challenge may be crucial in establishing the diagnosis. Future studies assessing the sensitivity and specificity of new diagnostic strategies to establish the presence of immediate or non-immediate reactions are required to better manage these patients.

## Consent

Written informed consent was obtained from the patients’ legal guardians for publication of this case report and any accompanying images. A copy of the written consents is available for review by the Editor-in-Chief of this journal.
